# The Effects of Local Police Surges on Crime and Arrests in New York City

**DOI:** 10.1371/journal.pone.0157223

**Published:** 2016-06-16

**Authors:** John MacDonald, Jeffrey Fagan, Amanda Geller

**Affiliations:** 1 Department of Criminology, University of Pennsylvania, Philadelphia, PA, United States of America; 2 Columbia Law School, Columbia University, New York, NY, United States of America; 3 Department of Sociology, New York University, New York, NY, United States of America; Johns Hopkins Bloomberg School of Public Health, UNITED STATES

## Abstract

The New York Police Department (NYPD) under Operation Impact deployed extra police officers to high crime areas designated as impact zones. Officers were encouraged to conduct investigative stops in these areas. City officials credited the program as one of the leading causes of New York City’s low crime rate. We tested the effects of Operation Impact on reported crimes and arrests from 2004 to 2012 using a difference-in-differences approach. We used Poisson regression models to compare differences in crime and arrest counts before and after census block groups were designated as impact zones compared to census block groups in the same NYPD precincts but outside impact zones. Impact zones were significantly associated with reductions in total reported crimes, assaults, burglaries, drug violations, misdemeanor crimes, felony property crimes, robberies, and felony violent crimes. Impact zones were significantly associated with increases in total reported arrests, arrests for burglary, arrests for weapons, arrests for misdemeanor crimes, and arrests for property felony crimes. Impact zones were also significantly associated with increases in investigative stops for suspected crimes, but only the increase in stops made based on probable cause indicators of criminal behaviors were associated with crime reductions. The largest increase in investigative stops in impact zones was based on indicators of suspicious behavior that had no measurable effect on crime. The findings suggest that saturating high crime blocks with police helped reduce crime in New York City, but that the bulk of the investigative stops did not play an important role in the crime reductions. The findings indicate that crime reduction can be achieved with more focused investigative stops.

## Introduction

Scholars have argued that changes in the tactics and management of police in the U.S. are a fundamental explanation for why crime rates have been low. Notable changes to police in the U.S. since the 1990s include the expansion of police forces, stronger accountability measures of command staff, the adoption of crime analytics, and the aggressive enforcement of misdemeanor and traffic laws [1–9]. These tactical and management innovations by police have been termed the “new policing” model [10].

The use of proactive police tactics to disrupt criminal activities is an essential element of the new policing model, and have been credited for why New York City (NYC) is now one of America’s safest cities [8].

Recent debates on the effect of the police on crime in NYC have centered on the use of investigative stops, and their role in maintaining the city’s low crime rate [11–14]. This approach also has been the most controversial, as investigative stops raise fundamental constitutional questions that have been the subject of government investigations [11] and numerous court cases [15–16].

In this paper, we examine the effects of Operation Impact, a signature New York Police Department (NYPD) program for over a decade, and a prototypical application of the “new policing” model that coupled police deployment with intensive investigative stop activity in high crime areas identified as impact zones. In the nearly two decades following NYC’s large crime decline of the 1990s [1, 17], scholars have continued to debate how much these policing tactics–including investigative stops–have sustained the low levels of crime in the city. Zimring [8] argues that the sustained deployment of extra police to high crime city blocks in NYC was responsible for the city’s crime reduction. Other research cites the increasing use of misdemeanor arrests as an essential ingredient of NYC’s crime reduction [18]. More recently, research has claimed that intensive use of investigative stops by the NYPD on high-crime street segments produced crime declines [13].

Prior research has not conclusively identified the long-term effects of police deployment or investigative stops on crime in NYC. Studies have examined either changes in arrests [5] or changes in investigative stops and crime [6, 11], without adequately accounting for reverse-causality (e.g., crime influences where arrests and stops are made). There is an extensive body of research that shows exogenous changes in police deployment and tactics caused by terror events [19–20] or warnings [21], police “crackdowns” [22], or through field experiments [23–24] reduce crime. Most of these studies, however, account for relatively short-term changes. With few exceptions [25], studies do not tell us whether there are enduring reductions in crime associated with surges in police deployment and the use of investigative stops. Also, few studies on police deployment and tactics estimate crime displacement. Donohue et al.’s [26] re-analysis of data from a previous study [19] on the effect of police deployment found that the crime reduction effect previously reported was due to the geographic displacement of crime.

While there is no consensus on which tactics are essential, the use of investigative stops combined with more stringent use of arrests for misdemeanor crimes (e.g., panhandling, public drinking and disorderly conduct) tend to be emphasized in the new policing model [27]. NYC is perhaps the most celebrated and closely studied city where changes in police tactics emphasized extra police deployment to high crime areas and the widespread use of investigative stops. Proponents of the use of these investigative stops by the NYPD have argued that the crime control returns were significant and uniquely attributable to this and other tactics implemented by the police [8, 13, 28].

Although there have been numerous accounts of the practice of investigative stops, few have carefully analyzed the effects of stops on crime. The most rigorous study to date by Weisburd and colleagues [13] using space-time interaction models estimated that weekly changes in investigative stops on street segments reduced crime by 2.0%. However, this study is not able to separate out the effects of extra deployment from the increase in investigative stops. There is the possibility that the visibility of extra police officers may have been as beneficial as the stops. Fagan [14] also estimated the effects of monthly stops, controlling for trends in two and six months before and after the current month, and found stops based on probable cause standards of criminal behavior were associated with a 5–9 percent decline in NYC crime in census block groups. Others have estimated the effects of investigative stops by assessing their impact on weapons seizure rates from police searches [29], finding that stops based on reasons that were more likely to lead to weapons seizures were less racially disparate and may have greater crime control benefits.

Knowledge about the effects of investigative stops on crime in NYC remains contradictory and incomplete. First, identification strategies in current research often lack counterfactuals of similarly situated places with high crime rates but that differ in stop activity. Nor do current studies fully avoid the potential of reverse causality, in that investigative stops can lead to crime through generating arrests and city ordinance violations. Second, the studies that use street segments as the unit of analysis [13] have to rely on the accuracy of the reporting of addresses and reconcile the ambiguity of how differences on two sides of the same street may be fundamental to stops and crime. For example, stops and crimes are often linked to a single address associated with a cluster of buildings, such as public housing projects, that are set back from the street face by anywhere from 25 feet to 50 yards. Crime markets in NYC are also likely to span more than one street segment [30]. The presence of vertical buildings in NYC also means that two sides of the same street may have completely different crime markets.

Together, research tells us quite a bit about the effects of short-term police surges on crime, but we know less about sustained deployment of extra police to high crime areas and little about the effect of investigative stops. Given the important claims of the efficacy of investigative stops that inform contemporary policing, and the contentious debates over its use in high crime areas, this study focuses on that specific tactic. NYC provides an ideal case to examine this issue. We examine the NYPD policing initiative of Operation Impact, which combined a police surge in deployment with intensified stop activity in high crime areas designated as impact zones, to identify the contributions of these activities to crimes and arrests. Operation Impact used essential features of the new policing model, including flooding high crime areas with more police officers and encouraging officers to conduct investigative stops.

This study builds on previous research in several ways. First, we exploit the temporal and spatial variation caused by Operation Impact to estimate the effect of police deployment and investigative stops on crimes and arrests at the census block group-level. Second, we examine the effects of police deployment on crime and arrests at the census block group-level while controlling for displacement to nearby areas. Third, we provide several robustness tests that examine how sensitive the results are the timing of when places were designated impact zones.

## Materials and Methods

In 2003, under Operation Impact the NYPD began a major change in its deployment practices by implementing the concept of an “impact zone”–a high crime area with specific boundaries that was designated to receive additional police fresh out of the police academy. In January 2003, the NYPD deployed roughly two-thirds of its police academy graduates—about 1,500 new police officers—to impact zones. In these areas, academy graduates were encouraged to engage in investigatory street stops and to enforce misdemeanor laws [31].

To identify areas as impact zones, police commanders nominated crime “hot spots” within their precincts that they thought would benefit from additional targeted resources. Using street-level crime data presented on maps, police crime analysts produced detailed statistical reports and recommended ways of refining the targeted areas. After discussions among local commanders and headquarters analysts, the Police Commissioner initially selected 24 areas with the highest rates of crime to receive extra police officers [31].

**Fig 1:**
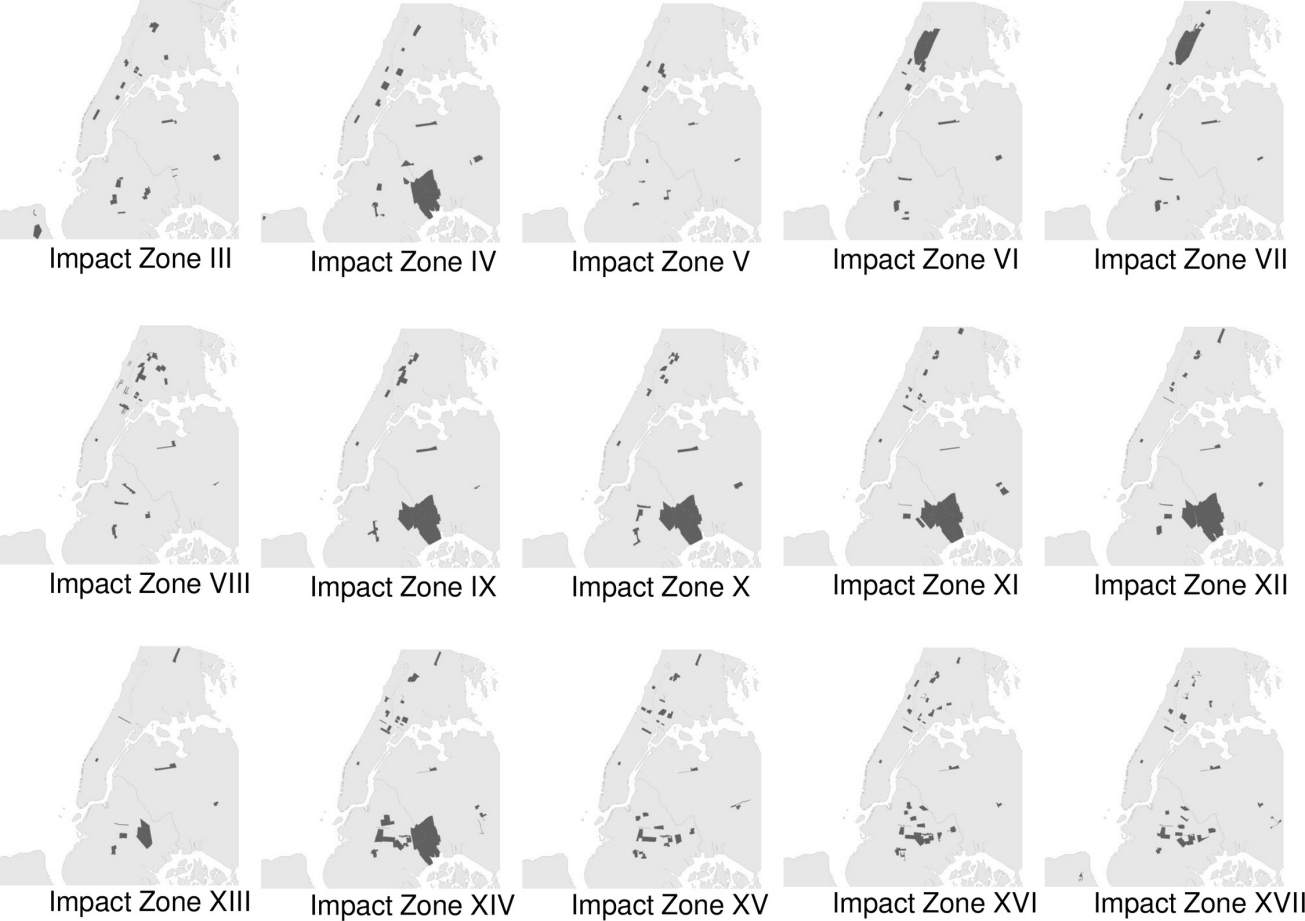
Rollout of Impact Zones 2004–2012.

By 2006, impact zones were present in 30 of the city’s 76 police precincts. Seventy-five precincts had at least one impact zone between 2004 and 2012. Impact zones were mostly located in high crime precincts where the majority of residents are Black and Latino. The precincts with the largest concentration of impact zones, for example, include East Harlem (23^rd^), Harlem (32^nd^), South and West Bronx (40^th^, 44^th^, 46^th^, and 52^nd^), and Brooklyn (70^th^, 75^th^, and 79^th^). Fig 1 shows a map of the location of impact zones and their rollout over time.

**Fig 2:**
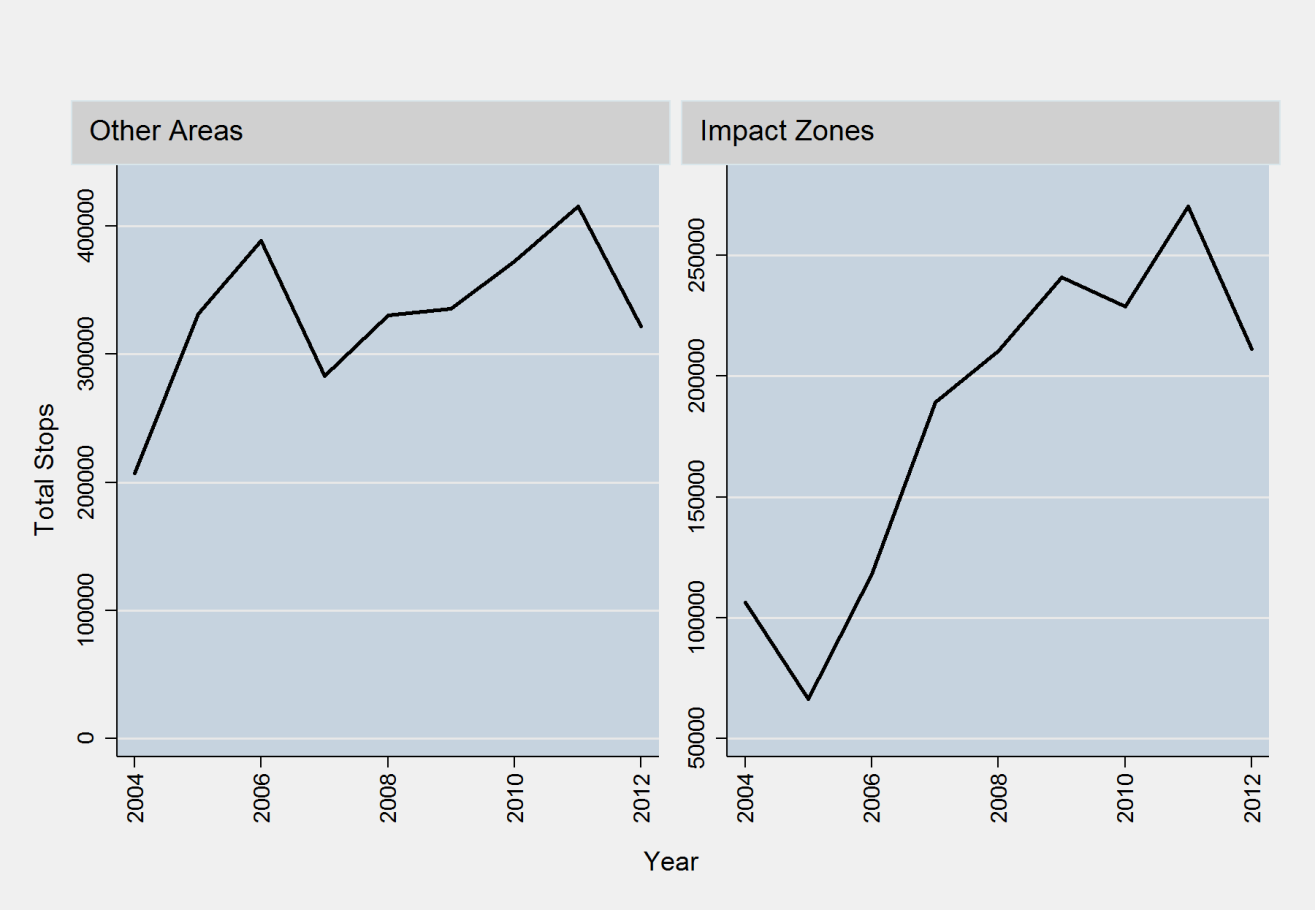
SQF Activity By Year.

Saturation of officers in impact zones produced higher stop rates per block (and per 100 crimes) than in other places in the city. Fig 2 shows the trend in stop rates by impact zones and the rest of the city. Stops rose by 14.2% in impact zone areas compared to 4.2% per year in other parts of the city. This estimate underscores the fact that impact zones both deployed more officers and generated more investigative stops.

The NYPD collected and geocoded reported crime complaints, arrests, and investigative stops during the time span (2004–2012) that encompasses the full implementation of Operation Impact. We geo-coded these incident data to the nearest census block group. We then aggregated the incident-level data to generate monthly census block-group counts of crimes, arrests, and stops for years 2004 to 2012. We use census block groups as the unit of analysis because the police designed impact zones using a wide circumference spanning multiple blocks, as well as clusters of public housing projects, that accompany crime markets. Thus, the census block group provides a closer approximation to the operation of police and crime markets than smaller geographic units like street segments. Aggregating the data to census block groups also minimizes NYPD mapping errors of placing crime, arrest, and stop locations at intersections or in the middle of the street when the street address is missing from officer reports.

We measure the total count of reported crimes and arrests. We also include separate counts for robbery, assault, burglary, weapons, misdemeanor offenses (e.g., criminal mischief, fraud, gambling, loitering, petty theft, and larceny), other felonies (e.g., escape 3 and forgery), drugs (e.g., dangerous drugs), property (e.g., grand larceny, burglary, and burglary tools), and violent felonies (e.g., homicide, rape, robbery, arson, felony assault, and kidnapping). We do not calculate rates of crime per population because such rates will be distortedly high in business areas of NYC, such as Times Square or Wall Street, which have daytime populations that far exceed their residential population [32]. We include an indicator for the police precinct where each block is located. Precincts are the administrative units that determine officer assignment. We also include measures for whether the census block group is located in an impact zone based on digital maps and shapefiles supplied by the NYPD.

Identifying the effect of Operation Impact on reported crimes is complicated because the police self-select the areas to become impact zones. To minimize this potential selection bias, we estimated the effect of impact zones on crimes and arrests using a difference-in-differences (DD) estimation. Under this specification, before and after changes in the monthly counts of crimes and arrests for those areas that become impact zones are compared to areas in the same police precincts that do not become impact zones. The first model (1) we estimate takes the form:

$$\log(Y_{it})=\;\beta_{0}+\;\beta_{1}D_{it}+{\alpha^{\prime }}_{precinct(i)}(t)$$ (1)

where *Y*_*it ~*_ Poisson (*λ*_*it*_) is the count of crimes or arrests in census block group *i* (n = 8,091) in month-year (= 2004–01,…, 2012–12) *t* of precinct *p*. A dummy variable of D (= 1) is assigned to a block group when it becomes an impact zone. *β*_*1*_ captures the treatment effect of becoming an impact zone. Model (1) also includes a fixed effect for each precinct-month-year (*α*) to capture trends that are common to all census block groups in the same precincts during the same month and year. This model in effect controls for precinct-level policy changes that are independent of Operation Impact.

The DD estimates will understate the effect of impact zones if enforcement in those areas creates spillovers, and overstate the effect of impact zones if they generate displacement to nearby blocks. To address this possible issue, we estimate a second model that takes the form:

$$\log(Y_{it})=\;\beta_{0}+\;\beta_{1}D_{it}+\beta_{2}N_{it}+{\alpha^{\prime }}_{precinct(i)}(t)$$ (2)

In model (2) a dummy variable N (= 1) is assigned to census block groups that are adjacent neighbors to areas that become impact zones. *β*_*2*_ captures the direct geographic displacement or spillover effect of census block groups becoming impact zones.

A key identifying assumption of models (1) and (2) is that impact zone assignments are independent of pre-existing trends in crime or arrests. If census block groups get assigned impact zones because of recent spikes in crimes or arrests, models (1) and (2) could overstate the effect of impact zones because of mean reversion. To minimize the potential effect of mean reversion of this form [33], we estimate a third model takes the form:

$$\log(Y_{it})=\beta_{0}+\sum_{k=1}^{2}\theta_{k}\;D_{it}+\sum_{k=1}^{2}\beta_{k}D_{it}+{\alpha^{\prime }}_{precinct(i)}(t)$$ (3)

Model (3) includes lags (θ) and leads (β) for the two months (t) before and after the implementation for those blocks that become impact zones [34]. The lags will absorb any influence due to movements in crimes or arrests in the two months just before adoption of impact zones. The lead parameters allow us to observe the effect of impact zones in the two months after their adoption.

Models 1 to 3 provide only estimates of the effect of impact zone presence, and do not identify the mechanism by which Operation Impact influences crime or arrest counts. Impact zones differ in size, location, and the number of officers deployed to them. To capture some differences in variation in the “dose” of proactive policing in impact zones we categorized the types of investigative stops conducted in each census block group. From stop data, we categorized stops as “probable cause” (P) when police officers checked the following indicators: (1) actions indicative of engaging in drug transaction; (2) actions indicative of violent crimes; or (3) “casing” victim or location. From stop data, we categorized stops as “general suspicion” (S) when police officers checked only the following indicators: (1) furtive movements, (2) fits descriptions, (3) carrying objects in plain view, (4) suspicious bulge, or (5) evasive actions [34]. From these classifications of stops, we estimate a fourth model that takes the following form:

$$\log(Y_{it})=\beta_{0}+\beta_{1}D_{it}+\beta_{2}P_{it}+\beta_{3}S_{it}+\beta_{4}DP_{it}+\beta_{5}{DS}_{it}+{\alpha^{\prime }}_{precinct(i)}(t)$$ (4)

Model (4) includes counts of the number of investigative stops based on indicators probable cause (denoted P) and general suspicion (denoted S). *β*_*4*_ captures the treatment effect of an impact zone based on the number of probable cause stops. *β*_*5*_ captures the treatment effect of an impact zone based on the number of general suspicion stops. Standard errors are clustered by precinct-month-year in all models to allow for dispersion and dependence common to precincts and time.

## Results

Table 1 shows the estimated effect of impact zones on crime and arrests from models 1 and 2. The results in the top rows show a negative effect of impact zones on crime. Model 1 implies that impact zones reduce the expected monthly count of total reported crimes by 12% (i.e., *e*^*-*.*124*^ = .88). Models that disaggregate by crime type show heterogeneity in effects. Weapons offenses and other felony offenses, which often are arrest-generated crimes, significantly increase in impact zones relative to other blocks in the same precinct at the same time of year. The increase in weapons offenses reflects primarily the seizure of knives from suspects. Gun possession arrests and gun seizures from street stops remained rare in NYC throughout this period, representing less than 1% of stops [35]. The largest proportional reduction in crimes occurs for burglary offenses, with an expected 46% reduction. Relatedly, the expected reduction in property felonies for burglary and theft is proportionally greater than violent felony offenses.

**Table 1:** Effect of Impact Zones on Crimes and Arrests.

*Crime*	*Total*	*Robbery*	*Assault*	*Burglary*	*Weapons*	*Misd*.	*Other*	*Drugs*	*Property*	*Violent*
							*Felony*		*Felony*	*Felony*
*Model 1*										
*Impact*	-0.124*	-0.157*	-0.131*	-0.611*	0.314*	-0.198*	0.614*	-0.026**	-0.296*	-0.120*
	(0.008)	(0.014)	(0.014)	(0.017)	(0.018)	(0.010)	(0.024)	(0.014)	(0.011)	(0.009)
*n =*	840,287	839,685	838,961	839,292	825,189	840,213	798,241	833,205	840,078	840,257
*Model 2*										
*Impact*	-0.149*	-0.139*	-0.148*	-0.663*	0.324*	-0.234*	0.629*	-0.030*	-0.332*	-0.129*
	(0.008)	(0.015)	(0.015)	(0.018)	(0.019)	(0.011)	(0.026)	(0.015)	(0.013)	(0.010)
*Neighbors*	-0.072*	0.050*	-0.049*	-0.158	0.026**	-0.105*	0.043**	-0.011	-0.111	-0.026*
	(0.007)	(0.012)	(0.012)	0.012	(0.014)	(0.010)	(0.025)	(0.011)	(0.011)	(0.007)
*n =*	840,287	839,685	838,961	839,292	825,189	840,213	798,241	833,205	840,078	840,257
*Mean*	5.43	.305	.192	.224	.124	2.24	.056	.381	.849	1.09
*Arrests*	*Total*	*Robbery*	*Assault*	*Burglary*	*Weapons*	*Misd*.	*Other*	*Drugs*	*Property*	*Violent*
							*Felony*		*Felony*	*Felony*
*Model 1*										
*Impact*	0.426*	-0.002	-0.017	0.387*	0.279*	0.298*	0.533*	-0.083*	1.174*	0.024
	(0.016)	(0.035)	(0.039)	(0.080)	(0.024)	(0.028)	(0.030)	(0.013)	(0.040)	(0.022)
*n =*	341,765	313,679	307,660	183,570	327,047	340,429	303,949	340,892	340,776	341,014
*Model 2*										
*Impact*	0.442*	0.060	-0.008	0.376*	0.295*	0.302*	0.562*	-0.093*	1.196*	0.051**
	(0.017)	(0.038)	(0.041)	(0.083)	(0.025)	(0.029)	(0.034)	(0.014)	(0.041)	(0.023)
*Neighbors*	0.049*	0.176*	0.025	-0.036	0.047**	0.011	0.086**	-0.029*	0.068**	0.078*
	(0.011)	(0.034)	(0.036)	(0.075)	(0.021)	(0.022)	(0.036)	(0.011)	(0.033)	(0.024)
*n =*	341,765	313,679	307,660	183,570	327,047	340,429	303,949	340,892	340,776	341,014
*Mean*	3.50	.100	.070	.021	.147	.264	.079	1.02	.524	.344

*p < .01

**p< = .05; Full sample represents 847,928 census block group month-years for crime and 341,962 census block group month-years for arrest.

Note: Total crime includes the sum of all 52 different crime categories consistently recorded. Total arrest includes the sum of all 65 arrest categories consistently recorded. Number drops in burglary arrest category because there are no burglaries in 3,495 census block groups. Arrest data are missing for 5 months of 2012. Robust standard errors are in parentheses.

The results from model 2 that include controls for adjacent neighbors show that impact zones still realize significant marginal crime reductions. The results also show that impact zone neighbors themselves see some crime reduction benefits. Model 2 implies that impact zones reduce the expected monthly count of total reported crimes in neighboring census block groups by 7% (i.e., *e*^*-*.*07*^ = .93). For specific crimes, the one exception is for robbery offenses, where there is evidence that the reduction in these offenses in impact zones is associated with an increase in adjacent census block groups. These findings suggest that impact zones may have displaced robberies to nearby areas.

The results for models 1 and 2 of arrests show that total arrests increase significantly in impact zones. Model 1 implies that the total amount of arrests increases by an expected 53%. The large increase in weapons, misdemeanor offenses, and burglary related arrests are the main drivers of the total arrest increase. Model 2 also shows a similar picture of impact zones increasing arrests. Total, robbery, weapons, and other felonies arrests also increase significantly in neighboring census block groups, suggesting some spillover of arrest actions to locations nearby impact zones.

**Fig 3:**
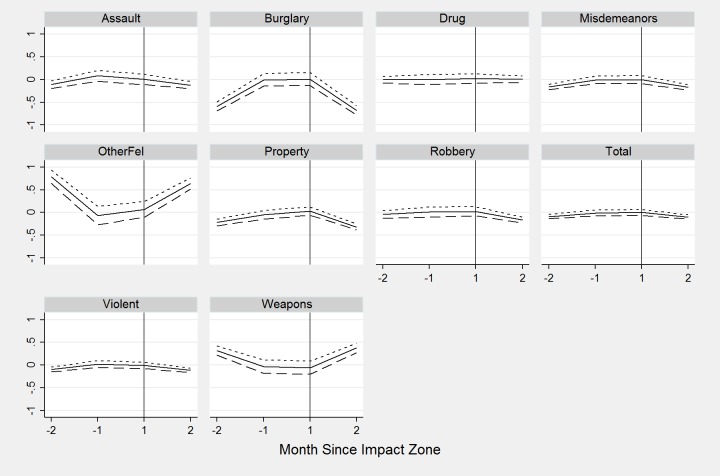
Event Study Estimates of Impact Zones on Crime. Month Since Impact Zone.

Fig 3 shows the results from model 3, which includes leads and lags for the two months before and after the formation of impact zones. S1 Table shows the complete results for all crime and arrest models. The total crime reduction attributable to impact zone designation is now 10% and sets in by the second month. The largest reductions occur for burglary, robbery, and property felony offenses, whereas weapons and other felony offenses related to arrests increases significantly. For each crime outcome, the coefficients are smaller in magnitude than model 1 and 2 suggesting that the formation of impact zones were partially determined by the crime rates in the two months preceding their implementation. For arrests, model 3 shows a significant increase of 61% that is similar in size to models 1 and 2. The significant increase in arrest counts are driven primarily by increases in arrests for burglary, weapons, misdemeanor offenses, and other property felonies offenses.

Table 2 shows the results from model 4 that estimates the dose-response of impact zones and probable cause (P) and general suspicion (S) stops. The results (not shown) indicate that both types of stops are more frequent where crimes are higher. However, the primary focus of this analysis is whether the timing of the impact zones and stop activity are associated with shifts in crime counts. For ease of interpretation, Table 2 only shows the coefficients from the interaction of stop types and impact zone implementation. The results indicate that increases in probable cause stops, after the formation of impact zones, are associated with reductions across several types of crime.

**Table 2:** Effect of Impact Zone Stops on Crime and Arrests.

*Crimes*	*Total*	*Robbery*	*Assault*	*Burglary*	*Weapons*	*Misd*	*Other*	*Drugs*	*Property*	*Violent*
							*Felony*			
*Model 4*										
PC*Impact	-0.011*	-0.010*	-0.009*	-0.015*	-0.011*	-0.012*	-0.017*	-0.014*	-0.011*	-0.010*
	(0.001)	(0.002)	(0.002)	(0.002)	(0.002)	(0.001)	(0.003)	(0.002)	(0.001)	(0.001)
NPC*Impact	0.002	-0.001	-0.003*	0.005*	-0.002	0.004*	-0.002	-0.002	0.002**	0.000
	(0.001)	(0.001)	(0.001)	(0.001)	(0.002)	(0.001)	(0.003)	(0.002)	(0.001)	(0.001)
*n =*	516,230	515,408	513,778	515,171	501,908	516,115	474,827	509,069	516,002	516,521
*Arrests*	*Total*	*Robbery*	*Assault*	*Burglary*	*Weapons*	*Misd*	*Other*	*Drugs*	*Property*	*Violent*
							*Felony*			
*Model 4*										
PC*Impact	-0.006*	0.000	0.002	-0.024*	-0.009*	-0.006*	-0.016*	-0.006*	-0.014*	0.002
	(0.002)	(0.004)	(0.004)	(0.010)	(0.002)	(0.002)	(0.003)	(0.001)	(0.003)	(0.002)
NPC*Impact	-0.006*	-0.010*	-0.011*	-0.001	-0.001	-0.006*	-0.003	-0.002	-0.011*	-0.009*
	(0.002)	(0.003)	(0.003)	(0.006)	(0.002)	(0.002)	(0.003)	(0.001)	(0.003)	(0.002)
*n =*	236,259	205,217	192,944	96,393	221,848	232,282	197,758	234,957	233,668	234,519

*p < .01

**p< = .05; Full sample represents 847,928 census block group month-years for crime and 341,962 census block group month-years for arrest.

Note: All models include parameters for Impact Zone, probable cause (PC), and non-probable cause (NPC) stops. Robust standard errors are reported in parentheses.

The estimates, however, suggest statistically significant results with little practical importance. The average number of total crimes in impact zones census block groups was 5.05 per month. This means that for 100 probable cause stops a month there would be roughly 3 fewer crimes [exp(-.01*100)*5.05]-[exp(-.01*0)*5.05] = -3.19]. There were 4.15 probable cause stops in an average month for impact zone census block groups. This indicates that there would need to be a five-fold increase in the number of probable cause stops made in an impact zone to avert more than one crime. By contrast, the increase in stops based on general suspicion has no consistent association with reductions in crimes. These stops appear to be both unproductive for reducing crime and may be constitutionally problematic. General suspicion stops are 50% more common on average than probable cause stops in impact zones.

The results for arrests show that stops for probable cause and general suspicion indicators are associated with fewer arrests in impact zones. These findings are consistent with other work showing that most investigative stops do not result in arrests [36].

### Robustness Checks

We conducted several robustness tests to examine the sensitivity of estimates to the selection of impact zones and the timing of their formation.

First, we re-estimated model 1, removing all census block groups that were initially part of Operation Impact Era 3 (January-June 2004), since we have no prior crime or arrest data for these areas. The results (not shown) are similar to those reported in Table 1, indicating that the findings are not sensitive to the inclusion of Impact Zone Era 3 locations, the baseline period when active offenders might have been caught off guard by the new surge in police.

Second, to address whether the estimates are at least partially attributable to autocorrelation in the timing of impact zones and the secular trends in crime and arrests citywide, we used a permutation test that randomly reassigned the timing of impact zone blocks 1,000 times and re-estimated model 1. The results show that neither crime nor the arrest estimates appear in the distributions of the permutation tests. The largest estimated reduction in crime is -.020 in the 1,000 shuffled timings compared to -.124 in our actual estimate. The largest estimated arrest increase is .035 in the 1,000 shuffled timings compared to .426 in our estimate. These findings confirm that autocorrelation in timing of impact zones is not driving the main results.

Third, models 1 to 4 all specify the changes in crime and arrests in impact zone block groups compared to other census block groups in the same precincts at the same month of a given year. To examine whether the effects observed in model 1 for crime and arrests were robust to the unit of comparison, we estimated a fifth model that included a fixed effect for each census block group and 4 cubic basis spline parameters for each precinct to capture the local smoothed time trend over the 96 months (Jan 2004-Dec 2012). The results from this analysis are similar but the effects are substantially smaller. Total reported crimes is negatively associated with impact zones, but is no longer statistically significant. Robbery, assault, and burglary remain negatively and significantly associated (p < .05) with impact zone formation. Weapons offenses also remain positively associated with impact zone formation. For arrests, the results also show that overall counts increase significantly with impact zones. The significant rise in weapons, misdemeanors, and property felony arrests is the leading cause of the overall increase in arrests. However, this model treats census blocks groups as independent units, when in fact census blocks groups within the same precincts become impact zones at the same time. Therefore, we think the original models (1–4) provide a closer approximation of the effects of impact zones on crimes and arrests.

## Conclusions

The U.S. Supreme Court in *Terry v*. *Ohio* [37] ruled police officers based on their experience and training had the power to stop, question, and frisk an individual when they had reasonable suspicion that a crime had just occurred, was in progress, or was about to take place. Officers were required to form “reasonable suspicion” based on specific, articulable, and individualized factors that were observable. The policy of relying heavily on investigative stops as a crime control program under Operation Impact created the conditions for a rigorous test of the effect of both targeted police deployment and investigative stops on crime. Operation Impact also presented an opportunity to test the claims that investigative stops were the likely cause of steadily declining crime rates over the past two decades in New York [8, 13].

The results suggest a complicated set of effects that present both good and bad news for concentrated police deployment and investigative stops as a crime reduction strategy in high crime areas. We found that Operation Impact had a statistically significant but relatively small association with a reduction in total crimes. The formation of impact zones had the largest effect on reducing robbery and burglary offenses. The data, however, do not distinguish a clear mechanism for this effect. The increase in probable cause-related stops after the formation of impact zone had the strongest association with reduced burglary and robbery reports, suggesting that physical presence of more police and enhanced apprehension may have generated a deterrent effect specific to those crimes. If officers were aware of the signs and indicia of crime, then the suppression of personal and property crimes by their presence and the use of investigative stops is a welcome byproduct of the surge of officers assigned to impact zones. However, probable cause-related stops were a relatively small fraction of the total number of investigative stops, suggesting that there were excess stops that had little crime suppression benefits. The scale of deployment and the level of stop activity suggest that this program may have been more productive if it placed more emphasis on probable cause stops more directly related to observable criminal activity. These findings are important for they suggest that more police activity and deployment to high crime areas can reduce criminal activity when constitutionally sound investigative tactics are used.

Operation Impact also appears to have significantly increased reported weapons and other felony-related offenses generated from arrests made by officers. The increase in weapons offenses are an artifact of arrests for this crime. This particular result is a positive outcome given the animating logic of the stop program generally, and Operation Impact in particular, to remove weapons from the streets.

Crime reduction is no doubt an important policy goal of police deployment, and investigative stops are an essential tool. There appears to have been some benefits of Operation Impact in reducing burglary and robbery crimes, and in increasing arrests for weapons. Deploying extra police to high crime areas and asking them to be vigilant appears to have some crime benefits—but only when vigilance is linked to articulable behaviors of suspected crimes occurring. Police interventions of the sort undertaken by Operation Impact should pay careful attention that increased vigilance does not come at the cost of extra intrusion and burdens on local residents that have no crime reduction benefit.

## Supporting Information

S1 Table Supplementary table showing results from model 4. (DOCX)
