# Supplementary material for: The Effects of Local Police Surges on Crime and Arrests in New York City
Source: PLoS One. 2016 Jun 16;11(6):e0157223. doi: 10.1371/journal.pone.0157223 (PMC4911104; doi:10.1371/journal.pone.0157223)
Supplement: S1 Table — (DOCX) [file pone.0157223.s001.docx]

# S1 Table: Lag and Lead Timing Results (Model 3)

| Crimes | Coef | stderr | t-stat | p-val | Time to Impact |
| --- | --- | --- | --- | --- | --- |
| Total | -0.090* | 0.024 | -3.769 | 0.000 | -2 |
| Total | -0.010 | 0.033 | -0.291 | 0.771 | -1 |
| Total | 0.001 | 0.033 | 0.025 | 0.980 | 1 |
| Total  *N=*840,287 | -0.098* | 0.024 | -4.167 | 0.000 | 2 |
| Robbery | -0.043 | 0.041 | -1.037 | 0.300 | -2 |
| Robbery | 0.010 | 0.056 | 0.181 | 0.856 | -1 |
| Robbery | 0.026 | 0.051 | 0.500 | 0.617 | 1 |
| Robbery  *N=*839,685 | -0.167* | 0.034 | -4.953 | 0.000 | 2 |
| Assault | -0.110* | 0.042 | -2.603 | 0.009 | -2 |
| Assault | 0.083 | 0.061 | 1.372 | 0.170 | -1 |
| Assault | 0.004 | 0.058 | 0.076 | 0.940 | 1 |
| Assault  *N=*838,961 | -0.121* | 0.041 | -2.919 | 0.004 | 2 |
| Burglary | -0.590* | 0.051 | -11.627 | 0.000 | -2 |
| Burglary | -0.006 | 0.069 | -0.086 | 0.931 | -1 |
| Burglary | 0.012 | 0.073 | 0.167 | 0.868 | 1 |
| Burglary  *N=*839,292 | -0.677* | 0.054 | -12.628 | 0.000 | 2 |
| Weapons | 0.322* | 0.051 | 6.277 | 0.000 | -2 |
| Weapons | -0.034 | 0.074 | -0.460 | 0.645 | -1 |
| Weapons | -0.059 | 0.074 | -0.795 | 0.427 | 1 |
| Weapons  *N=*825,189 | 0.377* | 0.053 | 7.173 | 0.000 | 2 |
| Misdemeanors | -0.162* | 0.031 | -5.290 | 0.000 | -2 |
| Misdemeanors | -0.007 | 0.043 | -0.152 | 0.879 | -1 |
| Misdemeanors | 0.000 | 0.044 | -0.002 | 0.999 | 1 |
| Misdemeanors  *N=*840,213 | -0.171* | 0.031 | -5.516 | 0.000 | 2 |
| OtherFel | 0.791* | 0.076 | 10.472 | 0.000 | -2 |
| OtherFel | -0.067 | 0.103 | -0.652 | 0.514 | -1 |
| OtherFel | 0.068 | 0.088 | 0.776 | 0.438 | 1 |
| OtherFel  *N=*798,241 | 0.640* | 0.063 | 10.145 | 0.000 | 2 |
| Drug | -0.005 | 0.037 | -0.140 | 0.889 | -2 |
| Drug | 0.002 | 0.055 | 0.033 | 0.974 | -1 |
| Drug | 0.022 | 0.052 | 0.429 | 0.668 | 1 |
| Drug  *N=*833,205 | 0.006 | 0.037 | 0.153 | 0.879 | 2 |
| Property | -0.220* | 0.037 | -5.985 | 0.000 | -2 |
| Property | -0.051 | 0.049 | -1.037 | 0.300 | -1 |
| Property | 0.032 | 0.046 | 0.701 | 0.483 | 1 |
| Property  *N=*840,078 | -0.319* | 0.034 | -9.348 | 0.000 | 2 |
| Violent | -0.097* | 0.027 | -3.533 | 0.000 | -2 |
| Violent | 0.021 | 0.038 | 0.555 | 0.579 | -1 |
| Violent | -0.010 | 0.035 | -0.279 | 0.780 | 1 |
| Violent  *N=*840,257 | -0.114* | 0.025 | -4.571 | 0.000 | 2 |

| Arrests | Coef | stderr | t-stat | p-val | Time to Impact |
| --- | --- | --- | --- | --- | --- |
| Total | 0.492* | 0.054 | 9.174 | 0.000 | -2 |
| Total | -0.017 | 0.073 | -0.230 | 0.818 | -1 |
| Total | 0.024 | 0.063 | 0.371 | 0.710 | 1 |
| Total | 0.482* | 0.044 | 10.928 | 0.000 | 2 |
| *N*=341,765 |  |  |  |  |  |
| Robbery | -0.061 | 0.103 | -0.592 | 0.554 | -2 |
| Robbery | 0.024 | 0.145 | 0.167 | 0.867 | -1 |
| Robbery | 0.002 | 0.149 | 0.014 | 0.989 | 1 |
| Robbery | 0.121 | 0.113 | 1.068 | 0.286 | 2 |
| *N*=313,679 |  |  |  |  |  |
| Assault | 0.031 | 0.107 | 0.290 | 0.772 | -2 |
| Assault | -0.074 | 0.149 | -0.497 | 0.620 | -1 |
| Assault | 0.145 | 0.148 | 0.980 | 0.327 | 1 |
| Assault | -0.095 | 0.108 | -0.872 | 0.383 | 2 |
| *N*=307,660 |  |  |  |  |  |
| Burglary | 0.855* | 0.241 | 3.540 | 0.000 | -2 |
| Burglary | -0.320 | 0.327 | -0.978 | 0.328 | -1 |
| Burglary | -0.150 | 0.318 | -0.472 | 0.637 | 1 |
| Burglary | 0.544* | 0.227 | 2.396 | 0.017 | 2 |
| *N*=183,570 |  |  |  |  |  |
| Weapons | 0.287* | 0.077 | 3.739 | 0.000 | -2 |
| Weapons | 0.007 | 0.101 | 0.069 | 0.945 | -1 |
| Weapons | -0.104 | 0.096 | -1.082 | 0.279 | 1 |
| Weapons | 0.401* | 0.073 | 5.484 | 0.000 | 2 |
| *N*=327,047 |  |  |  |  |  |
| Misdemeanors | 0.449* | 0.080 | 5.632 | 0.000 | -2 |
| Misdemeanors | -0.066 | 0.111 | -0.593 | 0.553 | -1 |
| Misdemeanors | -0.102 | 0.110 | -0.932 | 0.351 | 1 |
| Misdemeanors | 0.433* | 0.084 | 5.182 | 0.000 | 2 |
| *N*=340,429 |  |  |  |  |  |
| OtherFel | 0.715* | 0.103 | 6.934 | 0.000 | -2 |
| OtherFel | 0.008 | 0.135 | 0.060 | 0.952 | -1 |
| OtherFel | 0.175 | 0.108 | 1.625 | 0.104 | 1 |
| OtherFel | 0.490* | 0.079 | 6.228 | 0.000 | 2 |
| *N*=303,949 |  |  |  |  |  |
| Drug | -0.024 | 0.034 | -0.714 | 0.475 | -2 |
| Drug | -0.028 | 0.050 | -0.558 | 0.577 | -1 |
| Drug | 0.069 | 0.050 | 1.368 | 0.171 | 1 |
| Drug | -0.100* | 0.036 | -2.785 | 0.005 | 2 |
| *N*=340,892 |  |  |  |  |  |
| Property | 1.439* | 0.137 | 10.500 | 0.000 | -2 |
| Property | -0.169 | 0.177 | -0.953 | 0.341 | -1 |
| Property | -0.030 | 0.157 | -0.194 | 0.846 | 1 |
| Property | 1.220* | 0.107 | 11.458 | 0.000 | 2 |
| *N*=340,776 |  |  |  |  |  |
| Violent | 0.058 | 0.060 | 0.967 | 0.333 | -2 |
| Violent | -0.038 | 0.090 | -0.426 | 0.670 | -1 |
| Violent | -0.002 | 0.091 | -0.027 | 0.979 | 1 |
| Violent | 0.093 | 0.065 | 1.437 | 0.151 | 2 |
| *N*=341,014 |  |  |  |  |  |

*p<.01

Note: Robust standard errors are reported in parentheses.
